# NARD: whole-genome reference panel of 1779 Northeast Asians improves imputation accuracy of rare and low-frequency variants

**DOI:** 10.1186/s13073-019-0677-z

**Published:** 2019-10-22

**Authors:** Seong-Keun Yoo, Chang-Uk Kim, Hie Lim Kim, Sungjae Kim, Jong-Yeon Shin, Namcheol Kim, Joshua Sung Woo Yang, Kwok-Wai Lo, Belong Cho, Fumihiko Matsuda, Stephan C. Schuster, Changhoon Kim, Jong-Il Kim, Jeong-Sun Seo

**Affiliations:** 1 Precision Medicine Center, Seoul National University Bundang Hospital, 172 Dolma-ro, Seongnam, Bundang-gu, Gyeonggi-do 13605 Republic of Korea; 2 Precision Medicine Institute, Macrogen Inc., Seongnam, Republic of Korea; 3 Department of Biomedical Sciences, Seoul National University Graduate School, Seoul, Republic of Korea; 4 The Asian School of the Environment, Nanyang Technological University, Singapore, Singapore; 5 Singapore Centre for Environmental Life Sciences Engineering, Nanyang Technological University, Singapore, Singapore; 6 Department of Anatomical & Cellular Pathology and State Key Laboratory of Translational Oncology, The Chinese University of Hong Kong, Hong Kong, China; 7 Department of Family Medicine, Seoul National University Hospital, Seoul, Republic of Korea; 8 Center for Genomic Medicine, Kyoto University Graduate School of Medicine, Kyoto, Japan; 9 School of Biological Science, Nanyang Technological University, Singapore, Singapore; 10 Genomic Medicine Institute, Medical Research Center, Seoul National University, Seoul, Republic of Korea; 11 Gong-Wu Genomic Medicine Institute, Seoul National University Bundang Hospital, Seongnam, Republic of Korea

**Keywords:** Whole-genome sequencing, Reference panel, Genotype imputation, Northeast Asians, East Asians

## Abstract

Here, we present the Northeast Asian Reference Database (NARD), including whole-genome sequencing data of 1779 individuals from Korea, Mongolia, Japan, China, and Hong Kong. NARD provides the genetic diversity of Korean (*n* = 850) and Mongolian (*n* = 384) ancestries that were not present in the 1000 Genomes Project Phase 3 (1KGP3). We combined and re-phased the genotypes from NARD and 1KGP3 to construct a union set of haplotypes. This approach established a robust imputation reference panel for Northeast Asians, which yields the greatest imputation accuracy of rare and low-frequency variants compared with the existing panels. NARD imputation panel is available at https://nard.macrogen.com/.

## Background

During the past decade, the reference panels with population-scale whole-genome sequencing (WGS) have enabled the extensive human genetic research [1, 2]. They have played an imperative role in the genetic research, especially for genotype imputation in genome-wide association studies (GWAS). The most commonly used imputation panels were constructed by the 1000 Genomes Project Phase 3 (1KGP3) and Haplotype Reference Consortium (HRC) studies, which are publicly available for researchers. As genotype imputation is an essential step to increase the power of GWAS in a cost-efficient way, the confidence of imputed genotypes is the most important. To improve the quality of imputation in genetic studies, the large-scale population-specific reference panels with deep sequencing coverage are required. Accordingly, several research groups have generated large-scale WGS data to build their own population-specific reference panels [3–10].

Despite Northeast Asians account for 21.5% of worldwide population (http://www.worldometers.info/world-population), the majority of genetic studies and reference panels are biased to European ancestries [11]. There are some population-scale studies for building reference panels of Han Chinese (CHN), Japanese (JPN), Mongolians (MNG), and Koreans (KOR), but several issues, including public unavailability [6, 10, 12, 13], inadequate sequencing coverage [12, 14], small sample size [10, 15], and restriction to exonic regions [16, 17], need to be resolved for the solid imputation reference panel. Therefore, constructing a large-scale whole-genome reference panel covering the diverse population groups in Northeast Asia with deep sequencing coverage is still necessary to allow dense and accurate genotype imputation for the genetic research in these populations.

Here, we describe the Northeast Asian Reference Database (NARD), consisting of 1779 individuals from Korea, Japan, Mongolia, China, and Hong Kong. The goal of this study is to establish a high-quality population-specific reference panel for the genetic studies and precision medicine in Northeast Asia without the aforementioned issues.

## Construction and content

### Variant statistics

The NARD contains 1779 Northeast Asians including KOR (*n* = 850), JPN (*n* = 396), MNG (*n* = 384), CHN (*n* = 91), and Hong Kongese (HKG, *n* = 58) with deep (20× ≤, *n* = 834) or intermediate (10×~20×, *n* = 945) sequencing coverages (Additional file 1: Figure S1, Additional file 2: Table S1). Initially, WGS was performed on 1781 Northeast Asians, but two MNG samples with low variant count and an abnormal ratio of heterozygous to homozygous genotypes (Het/Hom) were discarded in the subsequent analysis (Additional file 1: Figure S2). We evaluated potential bias from inconsistent sequencing coverage of samples and found no significant correlation (Pearson correlation coefficient) between the sequencing depth and the number of variants: single nucleotide polymorphism (SNP; *R* = 0.15) and short insertion/deletion (indel; *R* = − 0.20). Also, transition to transversion (Ti/Tv) ratios were consistent across the samples (2.1 on average; Additional file 1: Figure S3). The Het/Hom ratios (1.4 on average; Additional file 1: Figure S4) and the number of loss-of-function variants (35.4 on average; Additional file 1: Figure S5) in the NARD were similar to those in East Asians from the 1KGP3 (1.3 and 36.9 on average for each). Also, 99.2% of the variants passed Hardy-Weinberg equilibrium (HWE) test (*P* > 1 × 10^−5^; Additional file 1: Figure S6).

In the NARD, a total of 40.6 million SNPs and 3.8 million indels were discovered, and 77.1% were singletons or rare variants (minor allele frequency [MAF] < 0.5%; Table 1). On average, 3.3 million SNPs and 0.3 million indels were found for each individual. We identified 15.4 million novel SNPs (37.8% of the total) in the NARD (Additional file 1: Figure S7a). Among them, 45.0% were specific to KOR, likely due to their large sample size, and 12.6% were found across populations (Additional file 1: Figure S7b). The majority of novel SNPs were singletons or rare variants and located in non-coding regions (Additional file 1: Figure S7c). We found the high integrity of our WGS variant call pipeline; the genotype concordance between WGS and Illumina Omni 2.5 M array of 86 CHN samples from the NARD was 99.6% (Additional file 2: Table S2).

**Table 1:** Total number of variants in 1779 individuals by MAF and functional category

Type	Frequency^a^	Number of variants	Functional variation								
			Protein coding region				Non-coding region				
			Silent/nonframeshift	Missense/frameshift	Stoploss/Stopgain	Unknown	Intronic	Intergenic	Splicing	UTR	ncRNA
SNP	Singleton	17,811,366	86,804	146,480	3722	2690	6,842,300	9,370,754	2110	247,422	1,109,084
	Rare	13,673,626	54,642	87,791	1658	1917	5,270,353	7,248,270	1363	164,492	843,140
	Low	3,430,315	12,753	15,710	232	428	1,299,727	1,851,373	245	38,673	211,174
	Common	5,727,339	17,886	15,981	151	729	2,049,372	3,228,994	159	53,221	360,846
	Total	40,642,646	172,085	265,962	5763	5764	15,461,752	21,699,391	3877	503,808	2,524,244
Indel	Singleton	1,402,707	3191	5068	157	129	558,772	717,182	517	27,748	89,943
	Rare	1,376,996	2733	2884	127	127	544,183	717,045	217	22,047	87,633
	Low	452,337	634	827	37	37	173,946	241,506	61	6444	28,845
	Common	569,436	422	369	18	89	207,132	317,135	145	7157	36,969
	Total	3,801,476	6980	9148	339	382	1,484,033	1,992,868	940	63,396	243,390

^a^Rare, MAF < 0.5%; low, 0.5% ≤ MAF < 5%; common, MAF ≥ 5%

### Ancestry composition of NARD

**Fig. 1:**
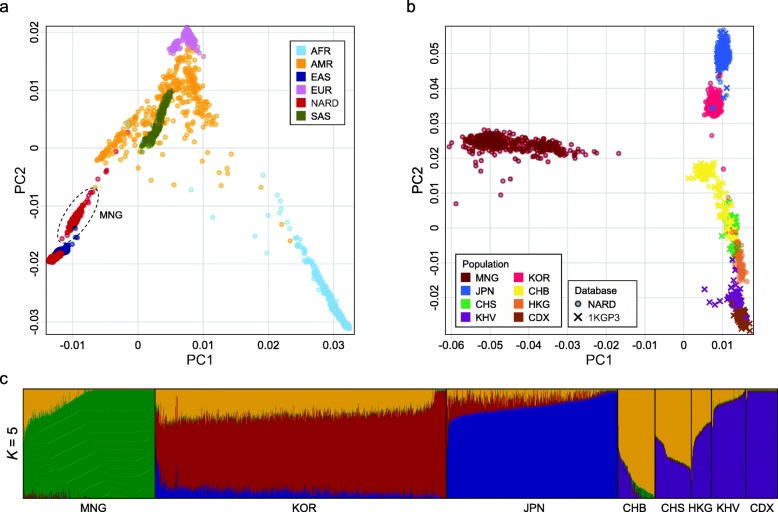
Ancestry composition of 1779 individuals in the NARD. **a** PCA of global populations from the NARD and 1KGP3. AFR, AMR, EAS, EUR, and SAS denote Africans, Americans, East Asians, Europeans, and South Asians, respectively. **b** PCA of Northeast and Southeast Asians from the NARD and 1KGP3. Japanese in Tokyo from the 1KGP3 were combined into JPN. CHN from the NARD were categorized into CHB and CHS. **c** Population substructure of Northeast and Southeast Asians with five ancestral components inferred by ADMIXTURE algorithm

We examined the ancestry composition of individuals in the NARD to illustrate how it covers the genetic diversity that was not present in other reference panels. From the principal component analysis (PCA) result of global human populations, individuals from the NARD were closely related to East Asians from the 1KGP3 as expected (Fig. 1a). MNG were separately clustered and positioned between East Asian and non-African populations as previously reported [10]. When we applied PCA to only Northeast and Southeast Asians, a clear population differentiation pattern was observed among them (Fig. 1b); MNG were most distinct from other populations based on PC1, and PC2 separated KOR, JPN, and mainland East Asians including Chinese Dai in Xishuangbanna (CDX), Han Chinese in Beijing (CHB), Han Chinese in Shanghai (CHS), HKG, and Kinh in Ho Chi Minh City (KHV). Interestingly, there were no overlapped samples between KOR and JPN except for a few outliers. This result implies that their ancestral compositions are distinctive enough to form separate clusters. Additionally, unsupervised ADMIXTURE analysis [18] supported the different ancestral components for each of KOR, MNG, JPN, and mainland East Asians (Fig. 1c). In the case of MNG, there were Buryats (BUR, *n* = 299), Khalkha Mongols (KHA, *n* = 73), and other Mongolians including Barga, Daringanga, Kazakh, Khoton, Uuld, Durvud, Khotogoid, and Zakhchin (OTH, *n* = 12). They were also genetically separated into BUR and KHA/OTH (Additional file 1: Figure S8). The results highlight that the NARD has the most diverse genetic compositions of Northeast Asian populations by adding the two ancestries, KOR and MNG, which have been underrepresented in public datasets such as the 1KGP3 panel.

### Evaluation of NARD imputation panel

**Fig. 2:**
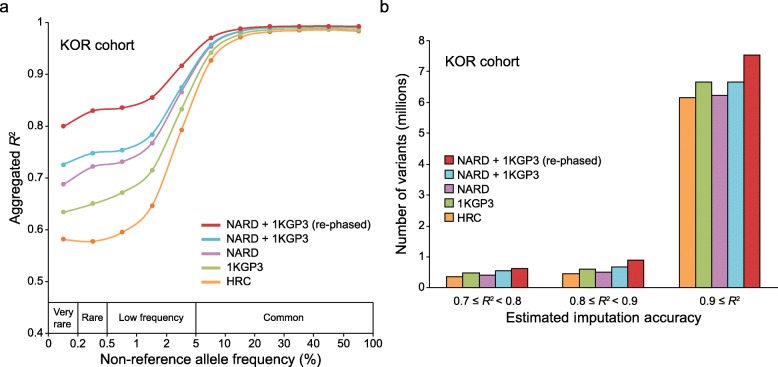
Imputation performance evaluation. **a** Imputation accuracy assessment using the five different reference panels. The pseudo-GWAS panel of 97 KOR was used for the imputation. The *x*-axis represents MAF of 850 KOR individuals from the NARD. The *y*-axis represents the aggregated *R*^2^ values of SNPs, which were calculated by the true genotypes and the imputed dosages. Only SNPs that were imputed across all panels were used for the aggregation of *R*^2^ values. **b** Number of imputed SNPs as a function of the estimated imputation accuracy and the types of imputation panel. This result was generated based on the *R*^2^ values that were estimated by Minimac3

To illustrate the robustness of the NARD as an imputation reference panel, we built a pseudo-GWAS dataset using an independent cohort of 97 unrelated KOR individuals [15, 19, 20] and simulated the genotype imputation analysis. It was generated from WGS data by masking the genotypes that were not included in the sites of Illumina Omni 2.5 M array. The imputation was conducted by Minimac3 [21] on the pre-phased SNPs using five types of reference panels: (1) NARD (*n* = 1779), (2) 1KGP3 (*n* = 2504), (3) HRC r1.1 (*n* = 32,470), (4) NARD + 1KGP3 (*n* = 4200), and (5) NARD + 1KGP3 (re-phased, *n* = 4200). To measure the imputation accuracy, we calculated the squared Pearson correlation coefficients (*R*^2^) between the true genotypes and the imputed dosages as a function of MAF in 850 KOR individuals from the NARD. The imputation performance of the NARD exceeded the 1KGP3 panel for every MAF bin (Fig. 2a). Notably, the HRC panel, with the largest sample size including individuals from the 1KGP3, showed poor performance compared with other panels. Since the low imputation accuracy of the HRC panel is inconsistent with the original investigation, we performed the same analysis using 24 unrelated French (FRA) individuals [22]. In contrast to a KOR cohort, we confirmed that the HRC panel produced the most accurate genotype dosages for an FRA cohort, and the NARD panel had poor suitability for Europeans (Additional file 1: Figure S9).

We then merged the NARD and 1KGP3 panels and performed re-phasing to enhance the imputation performance based on the previous studies [2, 5]. To combine the NARD and 1KGP3 panels without missing genotypes, we used the identical approach that was implemented in the UK10K and IMPUTE2 [5, 23]; we reciprocally imputed two panels using Minimac3 in order to statistically infer the missing genotypes in the NARD or 1KGP3 panels. Consistent with previous studies [3, 6, 8–10], combining the two panels showed more accurate imputation results compared with the NARD or 1KGP3 alone. Furthermore, we confirmed a large improvement of the imputation accuracy, particularly for very rare (MAF < 0.2%; *R*^2^ = 0.80), rare (0.2% ≤ MAF < 0.5%; *R*^2^ = 0.83), and low-frequency (0.5% ≤ MAF < 5%; *R*^2^ = 0.87) variants, when the haplotypes in the combined panel were re-phased by SHAPEIT3 [24]. In addition to measuring accuracy, we assessed the number of accurately imputed SNPs for each panel. For this analysis, we used the estimated *R*^2^ values measured by Minimac3, as it is the standard for the quality control procedure in GWAS [25, 26]. We found that the NARD + 1KGP3 (re-phased) panel produced the greatest number of high-confident SNPs (*R*^2^ ≥ 0.9) compared with other panels, especially 1KGP3 (*n* = 7.5 million versus 6.7 million), in concordance with the imputation accuracy (Fig. 2b).

We also illustrated the potential of the NARD + 1KGP3 (re-phased) as a reference panel for diverse Northeast Asians by performing additional imputation tests using independent cohorts of unrelated CHN and JPN individuals (*n* = 79 and 27, respectively) [27, 28]. For imputation accuracy measurement, we used MAF bins defined by 10,639 CHN and 3554 JPN individuals, respectively [13, 14]. In agreement with the imputation result of a KOR cohort, the NARD + 1KGP3 (re-phased) panel provided the most accurate genotype imputation on very rare (*R*^2^ = 0.71 and 0.84 for CHN and JPN cohorts, respectively), rare (*R*^2^ = 0.71 and 0.89 for CHN and JPN cohorts, respectively), and low-frequency (*R*^2^ = 0.81 and 0.91 for CHN and JPN cohorts, respectively) variants (Additional file 1: Figure S10a and S10b). The NARD + 1KGP3 (re-phased) panel also generated the largest number of accurately imputed genotypes compared with other panels, particularly 1KGP3 (*n* = 7.0 million versus 6.8 million and 6.6 million versus 6.2 million for CHN and JPN cohorts, respectively; Additional file 1: Figure S10c and S10d).

To investigate where the improvement of the NARD + 1KGP3 (re-phased) comes from, we divided the panel into the NARD (re-phased) and 1KGP3 (re-phased) and assessed the imputation accuracy separately. The NARD (re-phased) panel had slightly lower imputation power than the NARD + 1KGP3 (re-phased) panel, but greatly improved compared to the original NARD panel (Additional file 2: Table S4). Meanwhile, the 1KGP3 (re-phased) panel showed no improvement in the imputation accuracy compared to the original 1KGP3 panel.

We examined the underlying reasons for improved imputation performance caused by the re-phasing approach using identity-by-descent (IBD) analysis. It is known that phasing or genotype errors cause the gaps within the real IBD tracts; hence, the length of segments in phased genotype data tends to be shorter [29, 30]. Based on this aspect, we expected that haplotype correction is occurred by re-phasing, and it would extend the length of shared IBD segments among individuals. Therefore, we measured the shared large IBD segments (≥ 2 cM) between two individuals using the original (phased without 1KGP3) and re-phased haplotypes of the NARD. As a result, we confirmed the significant increase in length and number of shared IBD segments in re-phased haplotypes, which implies that the haplotype refinement in the NARD was achieved by the re-phasing process (Additional file 1: Figure S11).

### NARD imputation server

We developed a user-friendly web site to provide imputation service using the NARD + 1KGP3 (re-phased) panel for researchers (Additional file 1: Figure S12). Our web site provides the imputation process for a wide range of genotype data format including PLINK (ped and bed files paired with map and bim/fam files, respectively) [31], 23andMe (Mountain View, CA), AncestryDNA (Lehi, UT), and variant call format (VCF) [32]. Results are processed through the imputation pipeline consisting of four major steps: pre-processing, phasing, imputation, and post-processing. The pre-processing step checks the format and content validity of uploaded files and converts them into VCF files for the next steps. Depending on the format of uploaded files, PLINK and 23andMe/AncestryDNA files will be converted into VCF files using GotCloud [33] and BCFtools [34], respectively, based on hg19 reference coordinate. When the input files have multiple chromosomes, the server will automatically separate them into multiple files. The subsequent analyses proceed regardless of whether files have “chr” prefix in their contig names or not. The pre-processed data is phased using Eagle2 [35] or SHAPEIT2 [36], and Beagle5.0 [37] with or without a reference panel, respectively. Then, imputation is performed with Minimac4 (https://github.com/statgen/Minimac4). In the post-processing step, the output is assessed and provided as bgzip-compressed VCF and PLINK binary files. The server will provide the PLINK format with extra files containing predicted *R*^2^ values per variant for imputation quality check. Once imputation is finished, users will be notified by email and the result will be stored in the server for a week.

### NARD for variant interpretation

**Fig. 3:**
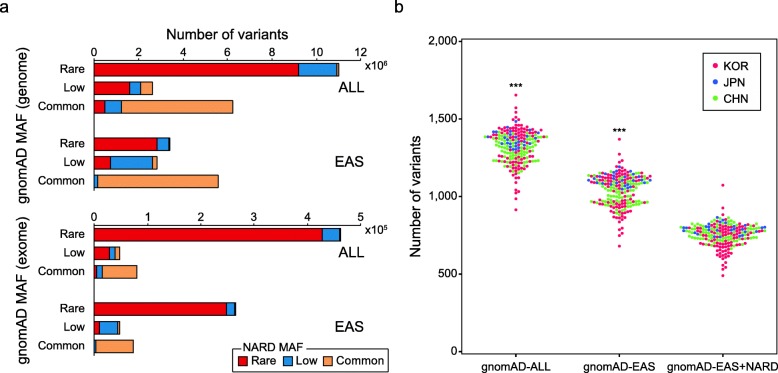
Variant interpretation using the NARD. **a** MAF differences of SNPs shared between the NARD and gnomAD. The y-axis denotes the MAF of SNPs in worldwide populations (ALL) or EAS from the gnomAD. Color represents the MAF of SNPs in 1779 Northeast Asians from the NARD. **b** Number of uncommon (MAF < 5%) protein-altering variants (missense, nonsense, frameshift, and splicing variants) after filtration using the gnomAD with/without NARD. Variant catalogue from the gnomAD (exome) was applied. ***P < 0.0001 by two-tailed Mann-Whitney *U* test (compared with gnomAD-EAS + NARD)

Filtering common variants based on the population allele frequency is the first step to identify rare disease-causing genes [38]. To examine the potential advantage of NARD for clinical variant interpretation, the frequencies of SNPs between the Genome Aggregation Database (gnomAD, 2.1.1 release) [39] and NARD were compared. We redefined the frequency of 1.8 million genome-wide SNPs that are rare in worldwide populations from the gnomAD (gnomAD-ALL) to low-frequency or common (MAF ≥ 5%). Moreover, 0.5 million rare genome-wide SNPs in East Asians from the gnomAD (gnomAD-EAS) were low-frequency or common variants in the NARD (Fig. 3a). We simulated rare disease variant discovery using 203 samples that were included in the three pseudo-GWAS panels for the imputation analysis. We applied variant filtering criteria (MAF < 5%) from the guidelines of the American College of Medical Genetics for the interpretation of sequence variants [40]. Notably, the number of protein-altering variants (missense, nonsense, frameshift, and splicing variants) was significantly reduced when the exome catalogues of gnomAD-EAS and NARD were jointly applied for variant filtration (Fig. 3b). This result represents that NARD could also contribute to the classification of pathogenic variant besides genotype imputation for the Northeast Asians.

### Whole-genome sequencing

For 1690 individuals of KOR, JPN, MNG, and HKG, we performed WGS using Hiseq X instrument (Illumina, San Diego, CA) based on the manufacturer’s instructions. We also used publicly available 91 CHN samples [41], which were sequenced by Illumina Hiseq 2000 instrument (Illumina, San Diego, CA). This cohort consists of YH cell line and samples from the HapMap and 1KGP3 with high sequencing depth (on average, 70×) [1, 42, 43].

### Variant discovery and refinement

Read alignment to the human reference genome (hg19) without any alternate contig, duplicate read removal, and joint calling of SNPs and indels were performed using Dynamic Read Analysis for GENomics platform (version 01.003.024.02.00.01.23004; http://edicogenome.com/dragen-bioit-platform/) with the following parameters: (i) creating gVCF: “--enable-map-align-output true,” “--remove-duplicates true,” “--enable-bam-indexing true,” “--enable-variant-caller true,” and “--vc-emit-ref-confidence GVCF,” and (ii) joint calling: “--enable-joint-genotyping true.” For indels, we discarded variants greater than 49 base pairs, which are generally defined as structural variants [20, 44]. Variant quality score recalibration (VQSR) was applied to raw variants based on the GATK’s best practice [45] with the parameters given below:

i) Annotations
  SNP: DP, QD, MQ, MQRankSum, FS, SOR
  Indel: DP, QD, MQ, MQRankSum, ReadPosRankSum, FS, SOR
ii) Truth set
  SNP: HapMap3.3 and 1KGP Omni2.5
  Indel: Mills & 1KGP gold standard
iii) Training set
  SNP: HapMap3.3, 1KGP Omni2.5 and 1KGP phase1 high confidence
  Indel: Mills & 1KGP gold standard and 1KGP phase 1
iv) Known set
  SNP: dbsnp138

SNPs and indels below 99% of truth sensitivity level from VQSR were initially filtered. Moreover, recalibrated variants were further filtered based on the following criteria: (i) located in the low complexity regions (LCRs), which were defined by the 1KGP3 study, (ii) genotype quality < 20, and (iii) read depth < 5. After these filtration processes, SNPs and indels were phased by SHAPEIT3 (version r884.1) which provides a fast population-scale phasing with low switch-error using the following model parameters: “--states 100,” “--window 2,” and “--effective-size 15000.”

### Variant discovery evaluation

We selected the 86 CHN samples in NARD with publicly available Illumina Omni 2.5 M array data from the 1KGP3, for validating variants in the NARD. A total of 1,664,330 SNPs were overlapped between NARD and Omni chip, excluding mitochondrial DNA and pseudoautosomal regions. The concordance is the cumulative sum of the matching alleles divided by the total number of loci multiplied by two, which is the maximum matching opportunity in diploid. The sex chromosomes in male are considered as diploids for this calculation.

### Variant annotation

All the SNPs and indels in this study were annotated by ANNOVAR based on the RefSeq gene definition [46, 47]. We annotated Kaviar [48], gnomAD, and The Single Nucleotide Polymorphism Database build 150 [27] for the classification of novel variants. For loss-of-function variant annotation, we implemented the Loss-Of-Function Transcript Effect Estimator (version 0.3-beta) [39] which is a plugin of Variant Effect Predictor [49] to remove low confidence annotations [50] with the following parameters: “--pick,” “--vcf,” “--cache,” “--offline,” and “--plugin LoF.” For the 1KGP3 dataset, we also removed the variants within LCRs.

### Hardy-Weinberg equilibrium calculation

We calculated HWE of variants in the NARD using VCFtools (version 0.1.12b) with “--hardy” option [32].

### Population structure analyses

We converted VCF files of bi-allelic autosomal SNPs from the NARD and 1KGP3 into PLINK format using GotCloud (version 1.75.5). Then, we merged the two panels by PLINK (version 1.9) and extracted SNPs with genotype rate equals to 100% and MAF ≥ 1% to remove the batch effect between the NARD and 1KGP3. Finally, we pruned SNPs with linkage disequilibrium (*R*^2^ > 0.1) within 50 base pairs sliding window using PLINK.

With this processed data, we carried out PCA with Genome-wide Complex Trait Analysis (version 1.91.3beta) [51] using (1) worldwide populations from the NARD and 1KGP3 and (2) Northeast and Southeast Asians from the NARD and 1KGP3, separately. We also applied the unsupervised ADMIXTURE algorithm (version 1.3) for ancestry estimation. The optimal cluster number was determined by comparing the cross-validation error rates of each *K* (Additional file 1: Figure S13). The results were visualized by Genesis (http://www.bioinf.wits.ac.za/software/genesis/).

### Imputation

For the imputation panel, the singleton variants in the NARD were excluded, because they are difficult to be imputed. To combine the NARD and 1KGP3 panels, we used the same approach as the UK10K and IMPUTE2; NARD-specific variants were imputed into the 1KGP3 using Minimac3 (version 2.0.1) and vice versa, then they were merged into a single reference panel. In addition, the combined panel was re-phased by SHAPEIT3 using the model parameters mentioned above with “--early-stopping” and “--cluster-size 4000” parameters. We kept variants that are not located in LCRs.

We separately processed 113 KOR, 79 CHN, 27 JPN, and 24 FRA individuals that are not included in the reference panels for imputation accuracy evaluation (Additional file 2: Table S3). Then, we discarded 16 related individuals from a KOR cohort. Unrelated sample selection was achieved by kinship estimation using KING [52]. Then, we extracted SNPs from sites on the Illumina Omni 2.5M array and monomorphic sites were excluded. As a result, 1,345,511, 1,320,123, 1,214,151, and 2,847,580 autosomal SNPs remained in the pseudo-GWAS panels of KOR, CHN, JPN, and FRA cohorts, respectively.

We performed imputation using Minimac3 with the five different types of reference panels. Imputation using the HRC panel was performed at the Michigan Imputation Server (https://imputationserver.sph.umich.edu). Before imputation, the haplotypes of individuals in the four cohorts were estimated using Eagle2 (version 2.3.2). After imputation, we extracted 4,352,921, 5,427,462, 48,431,56, and 5,419,512 SNPs in the four cohorts, which were imputed by all reference panels, and none of them were present with missing genotype in the non-masked dataset. The squared Pearson correlation coefficients (*R*^2^) were calculated between the imputed dosages and true genotypes, and those values were aggregated into 11 MAF bins to measure the imputation accuracy.

### IBD analysis

The shared IBD segments between two individuals were identified using RefinedIBD (version 12Jul18.a0b) with “length = 2.0” parameter [29]. To evaluate the effect of the re-phasing approach on haplotype correction, we performed this analysis using the original and re-phased haplotypes of the NARD which were phased without and with the 1KGP3 panel, separately. The short gaps and breaks (> 0.6 cM) between IBD segments were discarded using merge-ibd-segments utility program.

## Utility and discussion

Due to the cost-reduction and technological advancements in WGS, several groups have been focused on building the population-specific reference panels, especially for underrepresented populations in the conventional panels such as 1KGP3 [3, 4, 6–10, 13]. However, the Northeast Asian-specific reference panel with deep sequencing coverage and large sample size has been barely constructed and most of them are publicly unavailable. In this study, we integrated whole-genome sequence variants of 1779 Northeast Asian individuals to construct a reference panel, NARD; to resolve the uncertainty of genotype imputation along with the pre-existing panels; and to facilitate more comprehensive genetic analysis of Northeast Asians.

Genotype imputation accuracy is known to be affected by several factors, and one of the major determinants is the sample size of reference panel [5, 36]. Until now, most genotype imputations of Northeast Asians relied on the panels with large sample size [46–49], although the ancestries between the study population and reference panel are not matched. These panels showed lower imputation power, compared to the well-matched population-specific panels even with smaller sample size [3, 6–10, 53]. Considering the importance of population-specific reference panel, we generated a large-scale WGS dataset of KOR and MNG that were not included in the 1KGP3 panel. We confirmed that KOR and MNG were genetically differentiated from other East Asian populations. Therefore, the major ancestries in Northeast Asia are finally covered as population-scale by the NARD. In addition to the two populations, JPN, CHN, and HKG were also sequenced to increase the imputation power by the sample size effect and to build NARD as a reference panel that can be applied to diverse Northeast Asian populations.

Recently, the HRC panel was constructed using the genotypes of more than 30,000 individuals, mostly composed of European descent from various cohorts such as the 1KGP3 study. It is the largest publicly available reference panel, but previous studies demonstrated the poor imputation performance of this panel for CHN, admixed Africans, and Hispanic/Latino populations, even worse than the 1KGP3 panel [54, 55], and our analysis again supported this result. It is reasonably different from the original investigation of the HRC study because they only examined the imputation accuracy using European ancestry. The inconsistent results between the HRC study and others imply that several complex properties should be considered for achieving high-quality genotype imputation. It could be speculated that the population specificity between the reference panel and the individuals to be imputed would be occasionally more relevant factor than the size of reference panel. Therefore, the HRC panel might not be a gold standard for non-European populations. We believe that our new reference panel and analysis are valuable resources for researchers who want to achieve more accurate genotype imputation in Northeast Asians.

As previous studies yield further increment of the imputation accuracy from their population-specific panels by combining dataset of the 1KGP3 [3, 6, 8–10], we also confirmed the improvement of the imputation performance by combining the NARD and 1KGP3 panels using a fast and simple approach as described in the UK10K and IMPUTE2. However, there could be an issue regarding the uncertainty of imputed genotypes, since the missing genotypes in each panel were statistically estimated. Referring to the HRC study, calculating genotype likelihood of each variant using the individual BAM files would improve the uncertainty of these genotypes, if the sequencing coverages are sufficient. After merging the NARD and 1KGP3, we enhanced the power of the combined panel by applying the re-phasing strategy. It is an advanced process that has not been applied in most of previous studies [3, 6, 8–10], but the HRC study has shown further improvement of the imputation accuracy with this approach. Based on this strategy, the NARD + 1KGP3 (re-phased) panel produced more accurately imputed genotype dosages, especially for uncommon variants (MAF < 5%), than the NARD + 1KGP3 panel. This might be due to haplotype correction with the assistance of the haplotypes in the 1KGP3 panel.

## Conclusions

In summary, we generated a large-scale reference panel for Northeast Asians, which will be a highly valuable resource to resolve a persistent deficiency of Asian genome data. We believe that our efforts will remarkably contribute to precision medicine in Northeast Asia.

## Supplementary information

**Additional file 1: Figure S1.** Geographic map of the study area in the NARD. **Figure S2.** Correlation between the sequencing depth and number of variants. **Figure S3.** Transition to transversion ratio of the populations in the NARD. **Figure S4.** Heterozygous to homozygous ratio of the global populations. **Figure S5.** Number of loss-of-function variants. **Figure S6.** Hardy-Weinberg Equilibrium test of variants in the NARD. **Figure S7.** Novel variant statistics. **Figure S8.** Differential genetic composition of the two MNG groups. **Figure S9.** Imputation performance evaluation of FRA individuals. **Figure S10.** Imputation performance evaluation of CHN and JPN individuals. **Figure S11.** Length distribution of shared IBD tracts between the two individuals in each population. **Figure S12.** The flow chart of the pipeline consisting of four major steps for NARD imputation server. **Figure S13.** The cross-validation error inferred by ADMIXTURE algorithm.

**Additional file 2: Table S1.** Variant statistics. **Table S2.** Variant accuracy. **Table S3.** Data source for imputation analysis. **Table S4.** Imputation performance according to types of reference panel.

## Data Availability

Raw sequence data is protected and cannot be released to the public due to compromise of participant confidentiality and privacy. Alternatively, genotype imputation can be freely performed at the NARD imputation server for the academic purpose (https://nard.macrogen.com/). Researchers can download MAF data from the NARD as a VCF file (https://nard.macrogen.com/download/NARD_MAF.hg19.zip). The hg38 version of MAF data liftovered by CrossMap (version 0.3.6) [56] is also available (https://nard.macrogen.com/download/NARD_MAF.hg38.zip).

## Abbreviations

NARD: Northeast Asian Reference Database
GWAS: Genome-wide association study
1KGP3: 1000 Genomes Project Phase 3
WGS: Whole-genome sequencing
HRC: Haplotype Reference Consortium
CHN: Han Chinese
JPN: Japanese
MNG: Mongolians
KOR: Koreans
HKG: Hong Kongese
Het/Hom: Heterozygous to homozygous genotypes
SNP: Single nucleotide polymorphism
Indel: Short insertion/deletion
Ti/Tv: Transition to transversion
HWE: Hardy-Weinberg equilibrium
MAF: Minor allele frequency
PCA: Principal component analysis
CHB: Han Chinese in Beijing
CHS: Han Chinese in Shanghai
KHV: Kinh in Ho Chi Minh City
BUR: Buryats
KHA: Khalkha Mongols
OTH: Other Mongolians
FRA: French
IBD: Identity-by-descent
VCF: Variant call format
gnomAD: Genome Aggregation Database
gnomAD-ALL: Worldwide populations from the gnomAD
gnomAD-EAS: East Asians from the gnomAD
VQSR: Variant quality score recalibration
LCR: Low complexity region
