# Supplementary material for: NARD: whole-genome reference panel of 1779 Northeast Asians improves imputation accuracy of rare and low-frequency variants
Source: Genome Med. 2019 Oct 22;11:64. doi: 10.1186/s13073-019-0677-z (PMC6805399; doi:10.1186/s13073-019-0677-z)
Supplement: Supplementary file 1 — Additional file 1: Figure S1. Geographic map of the study area in the NARD. Figure S2. Correlation between the sequencing depth and number of variants. Figure S3. Transition to transversion ratio of the populations in the NARD. Figure S4. Heterozygous to homozygous ratio of the global populations. Figure S5. Number of loss-of-function variants. Figure S6. Hardy-Weinberg Equilibrium test of variants in the NARD. Figure S7. Novel variant statistics. Figure S8. Differential genetic composition of the two MNG groups. Figure S9. Imputation performance evaluation of FRA individuals. Figure S10. Imputation performance evaluation of CHN and JPN individuals. Figure S11. Length distribution of shared IBD tracts between the two individuals in each population. Figure S12. The flow chart of the pipeline consisting of four major steps for NARD imputation server. Figure S13. The cross-validation error inferred by ADMIXTURE algorithm. [file 13073_2019_677_MOESM1_ESM.pdf]

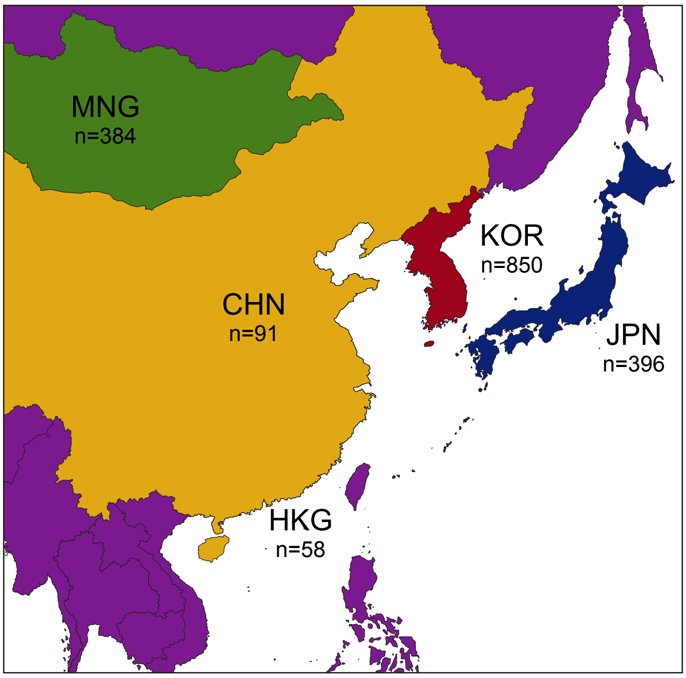

Fig. S1. Geographic map of the study area in the NARD.

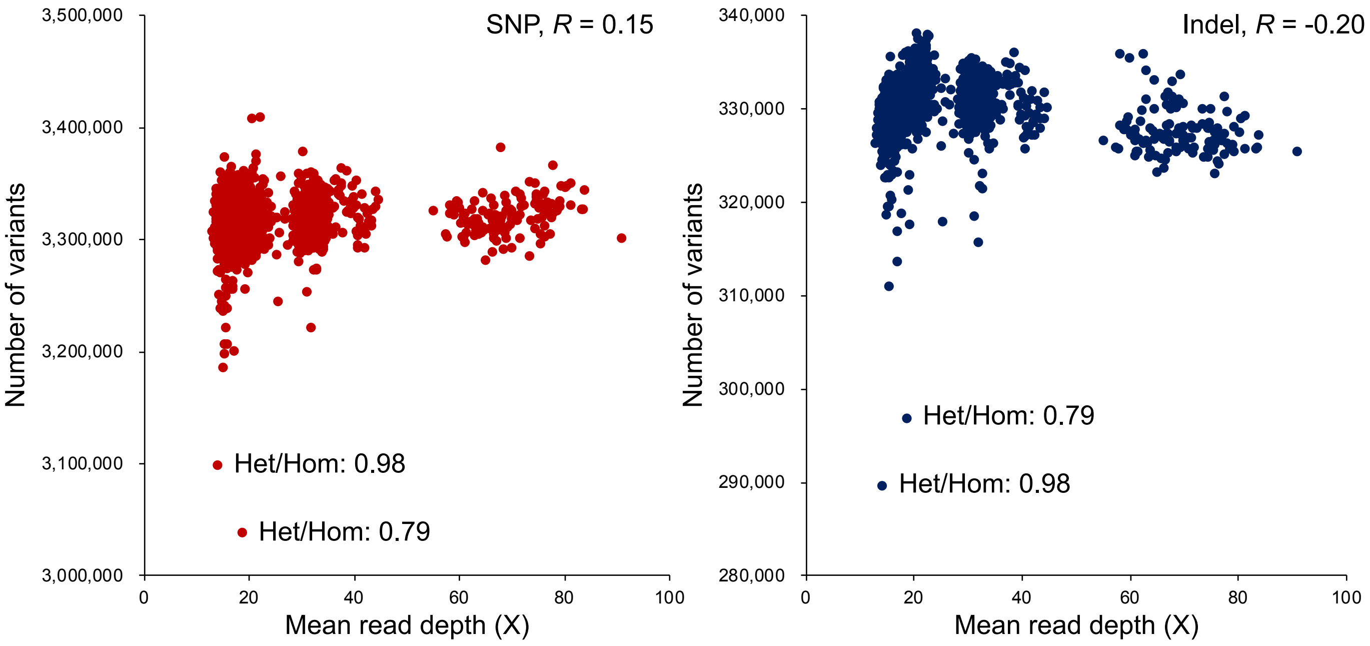

Fig. S2. Correlation between the sequencing depth and number of variants. Pearson correlation coefficient (*R*) was calculated excluding the two samples with an abnormal Het/Hom ratio.

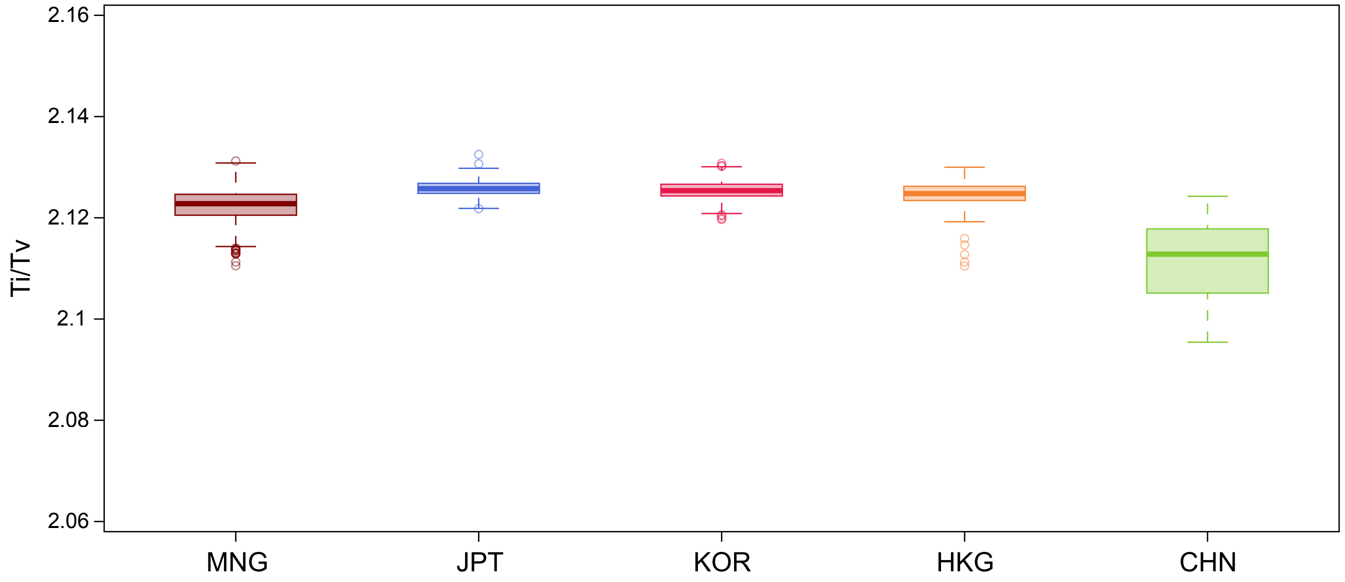

Fig. S3. Transition to transversion ratio of the populations in the NARD.

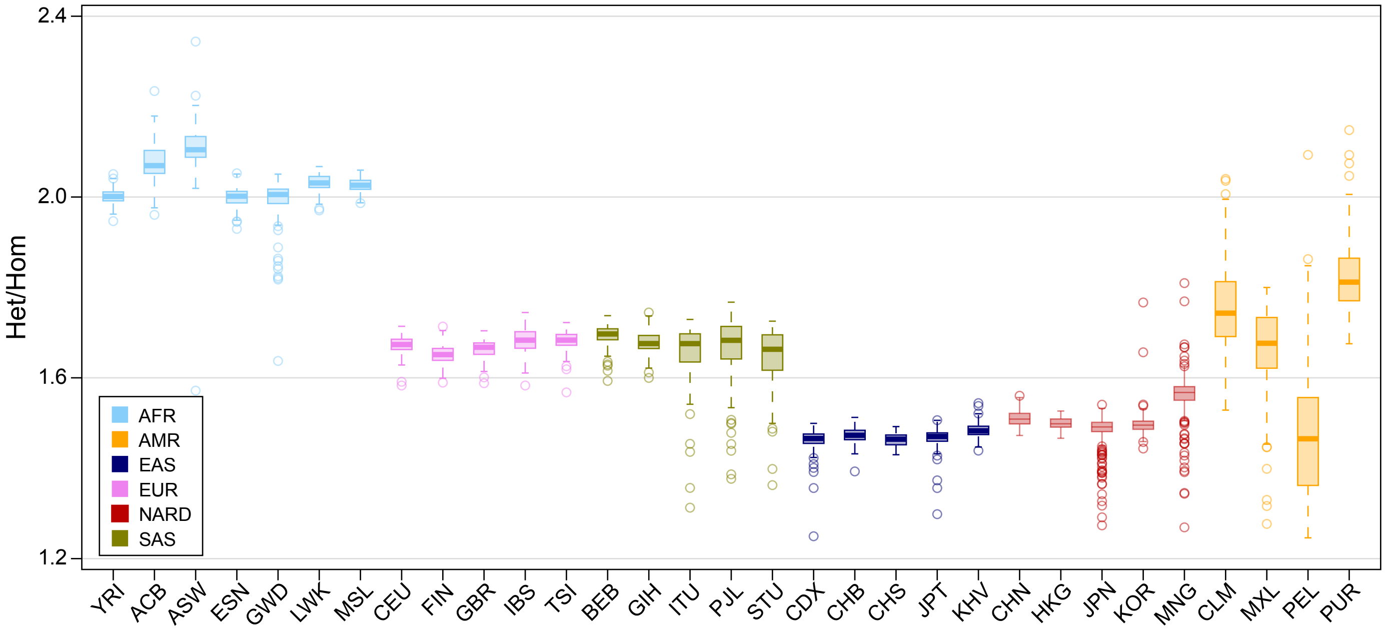

Fig. S4. Heterozygous to homozygous ratio of the global populations.

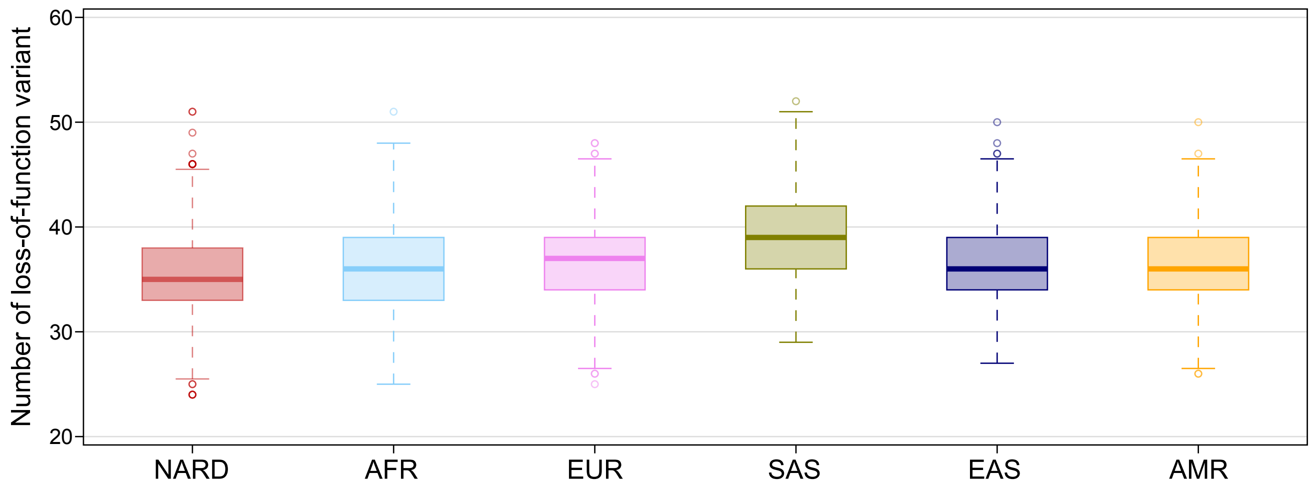

Fig. S5. Number of loss-of-function variants. The 1KGP3 dataset was divided into five super population code.

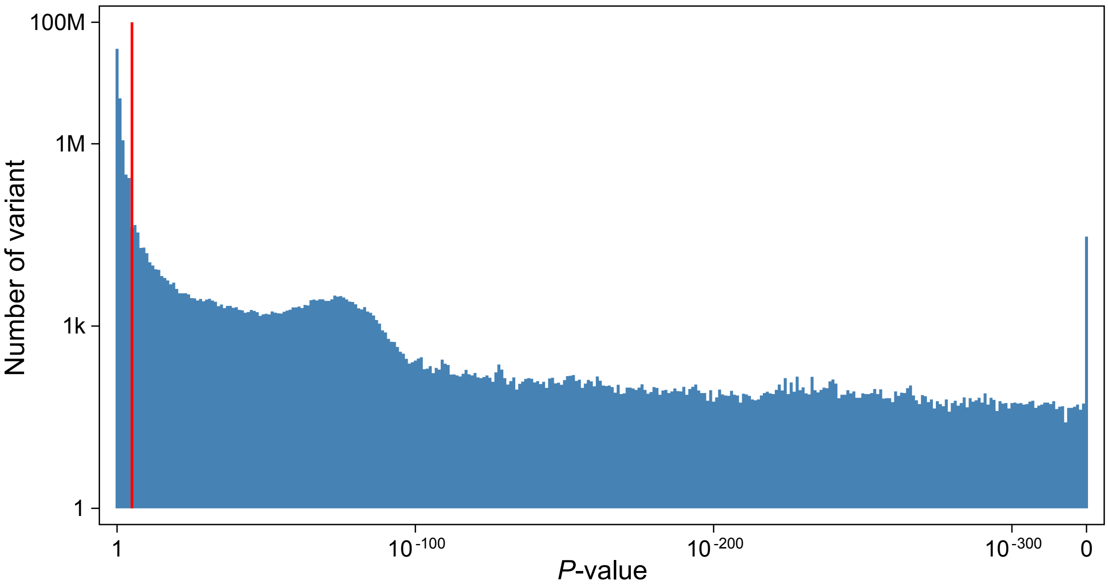

Fig. S6. Hardy-Weinberg Equilibrium test of variants in the NARD. Red line indicates the significance threshold (*P*-value = $1x10^{-5}$).

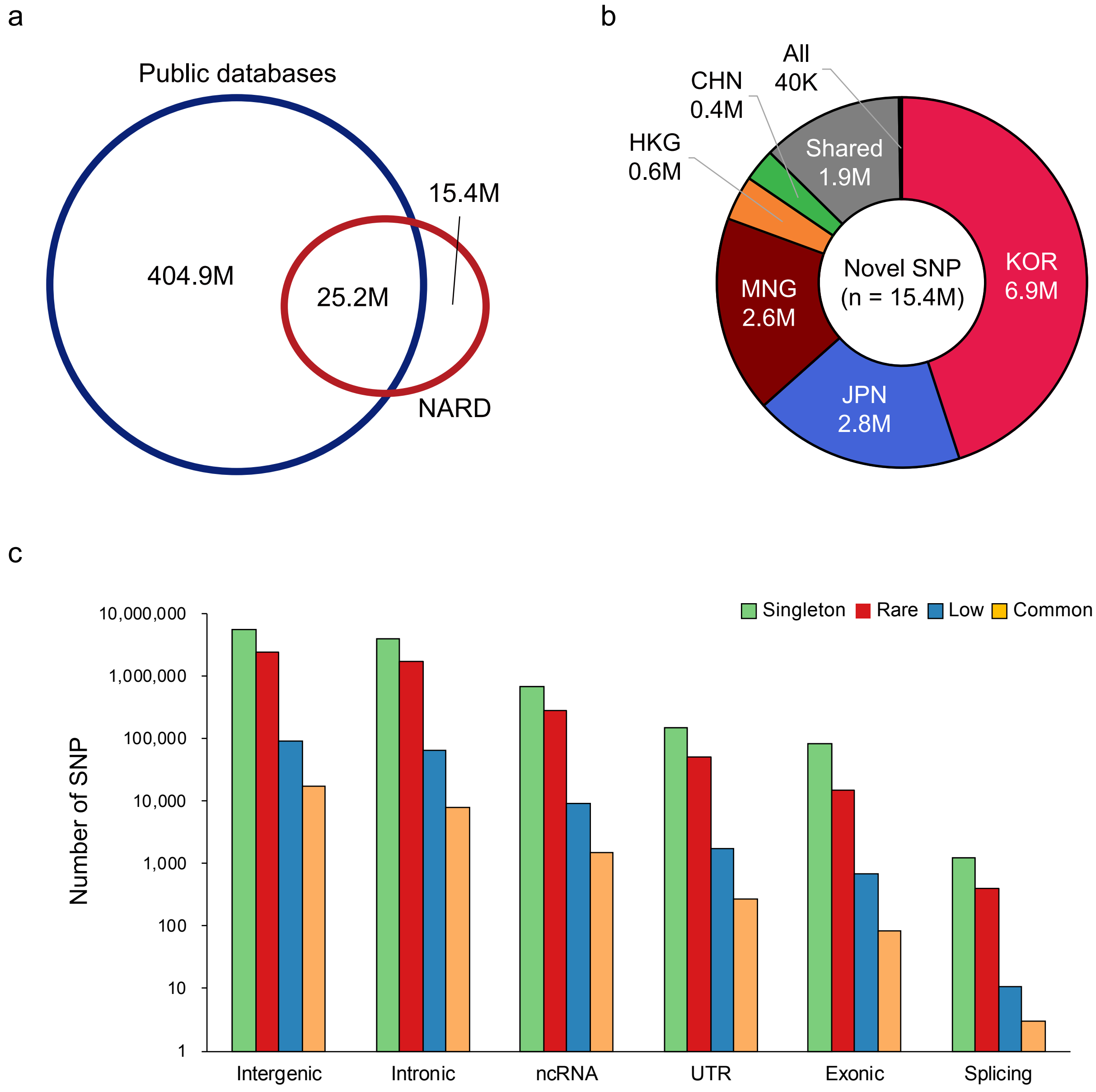

Fig. S7. Novel variant statistics. a, Number of novel SNPs that were not identified in elsewhere. Public databases include Kaviar, gnomAD (2.1.1 release), and dbSNP150. **b,** Distribution of novel SNP per population. Novel SNPs found in multiple and all populations were included in ‘Shared’ and ‘All’, respectively. **c**, Distribution of novel SNPs based on RefSeq gene definition.

a

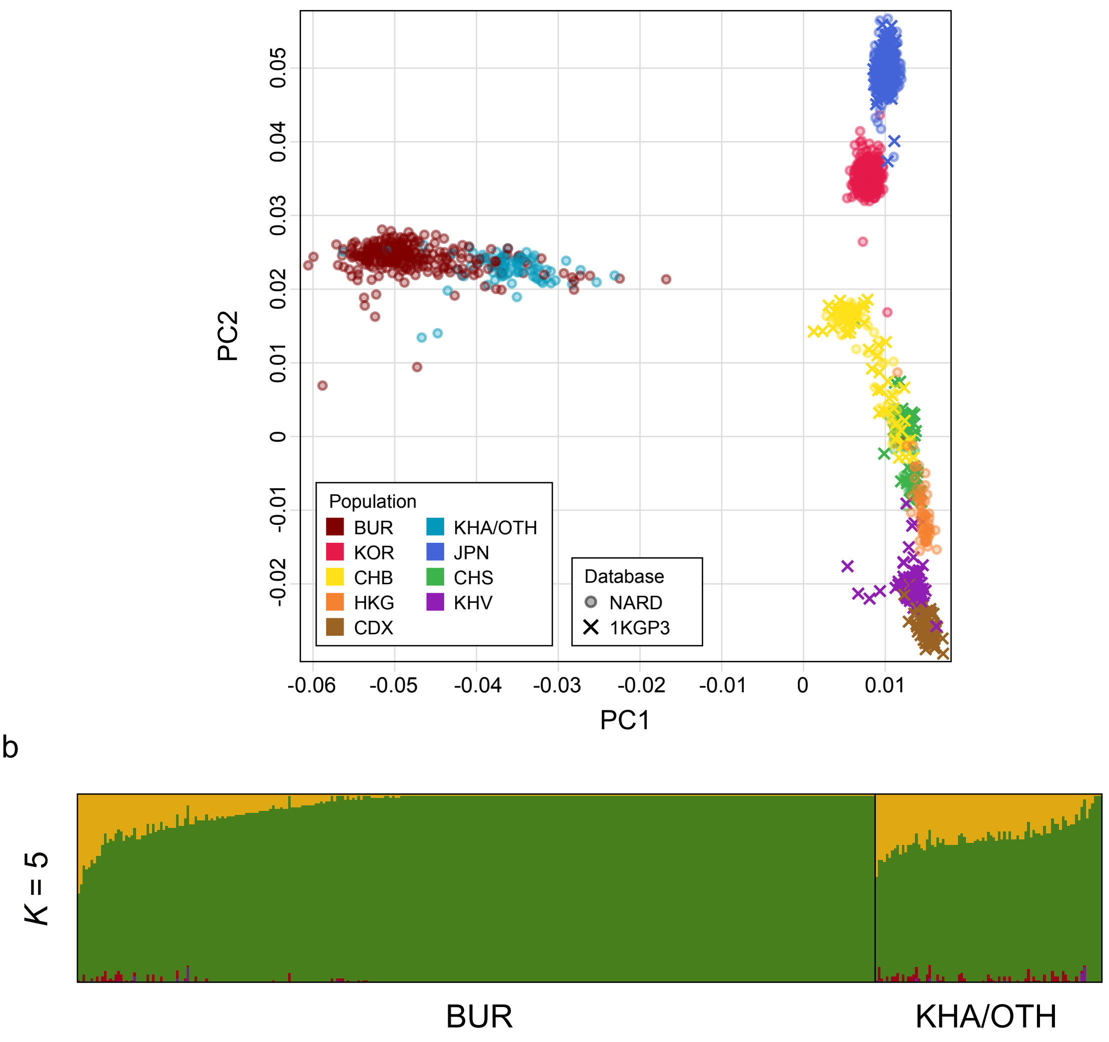

Fig. S8. Differential genetic composition of the two MNG groups. a, PCA of Northeast and Southeast Asians from the NARD and 1KGP3. MNG population is divided into Buryats (BUR) and Khalkha Mongols (KHA)/ Others (OTH). **b,** Population substructure of MNG with five ancestral components inferred by ADMIXTURE algorithm.

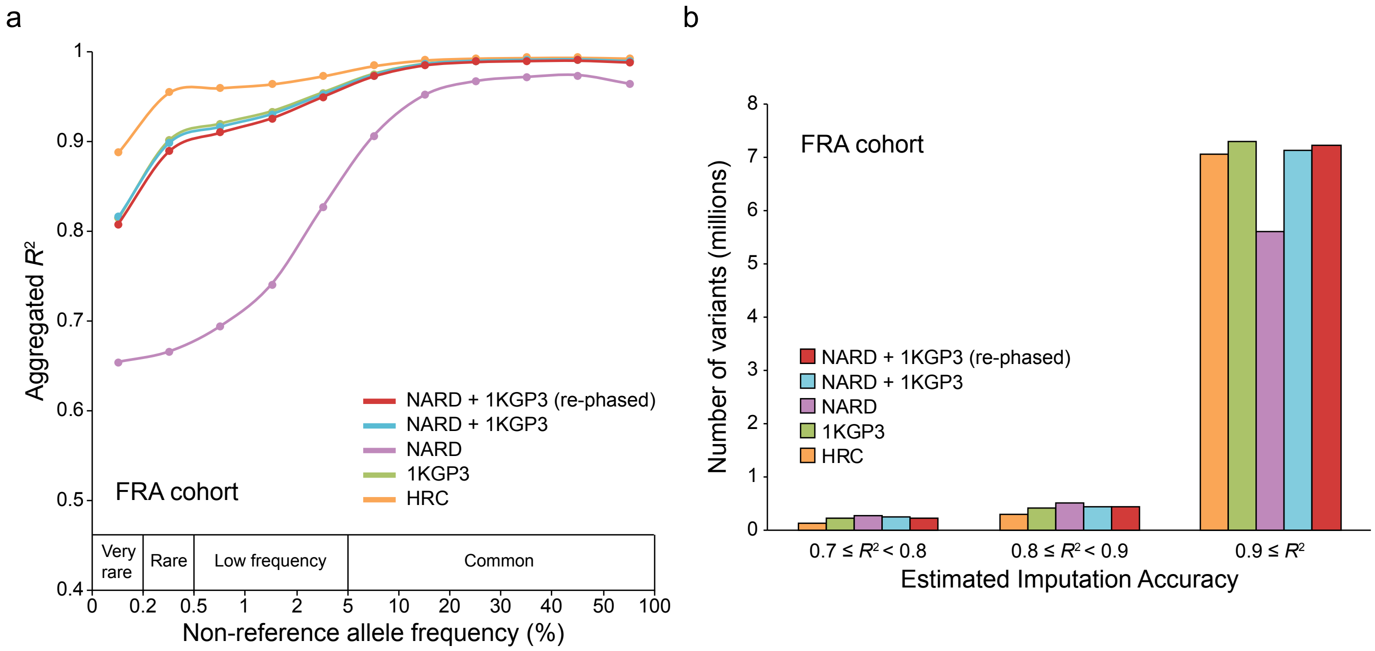

Fig. S9. Imputation performance evaluation of FRA individuals. a, imputation accuracy assessment using the five different reference panels. The pseudo-GWAS panel of 24 FRA was used for the imputation. The x-axis represents MAF of 7,718 non-Finnish Europeans individuals from the Genome Aggregation Database (2.1.1 release). The y-axis represents the aggregated R^2 values of SNPs, which were calculated by the true genotypes and the imputed dosages. Only SNPs that were imputed across all panels were used for the aggregation of R^2 values. **b,** Number of imputed SNPs as a function of the estimated imputation accuracy and the types of imputation panel. This result was generated based on the R^2 values that were estimated by Minimac3.

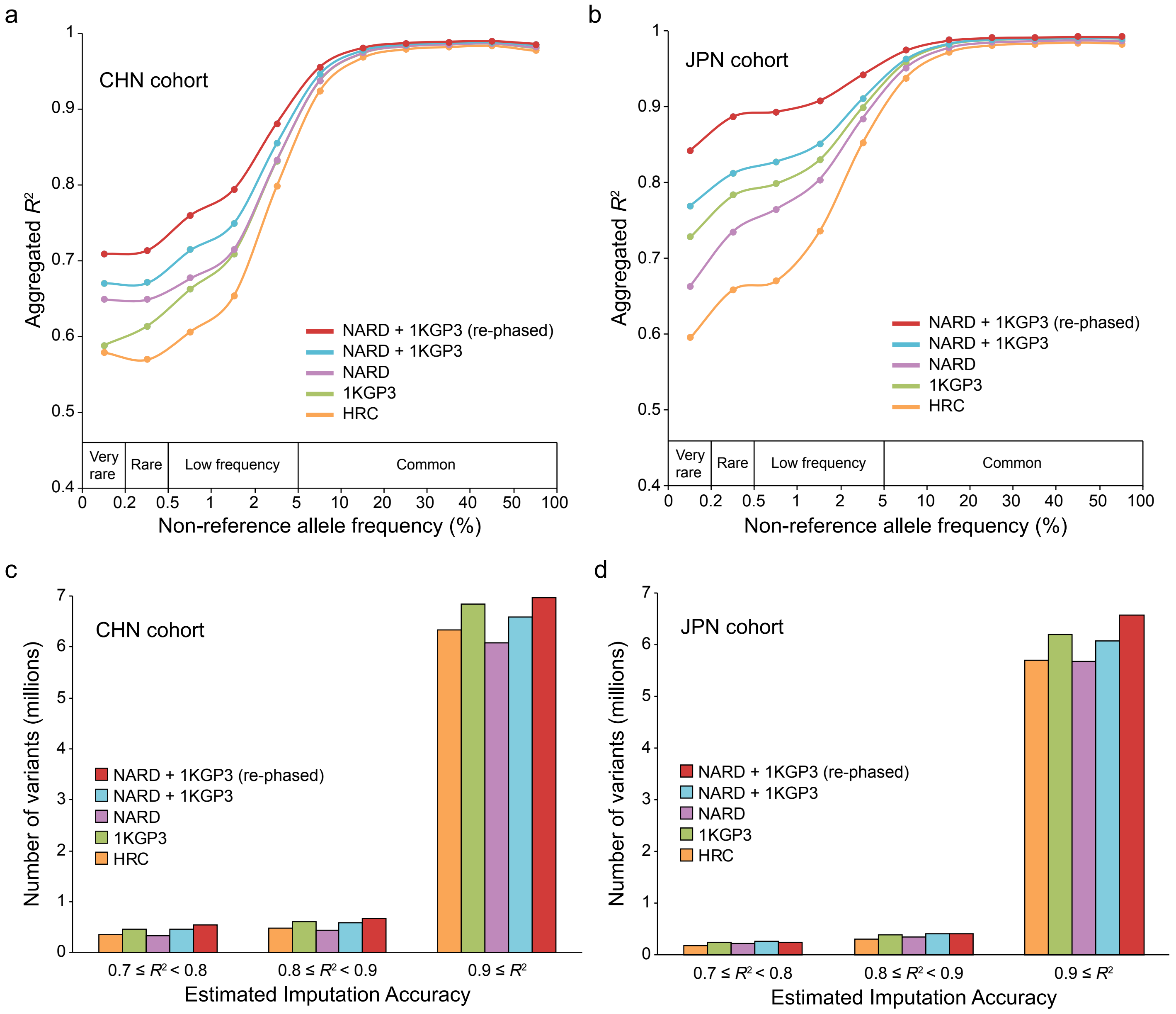

Fig. S10. Imputation performance evaluation of CHN and JPN individuals. a-b, imputation accuracy assessment using the five different reference panels. The pseudo-GWAS panel of 79 CHN and 27 JPN individuals was used for the imputation. The x-axis represents MAF of 10,639 CHN and 3,554 JPN individuals, respectively. The y-axis represents the aggregated R^2 values of SNPs, which were calculated by the true genotypes and the imputed dosages. Only SNPs that were imputed across all panels were used for the aggregation of R^2 values. **c-d,** Number of imputed SNPs as a function of the estimated imputation accuracy and the types of imputation panel. This result was generated based on the R^2 values that were estimated by Minimac3.

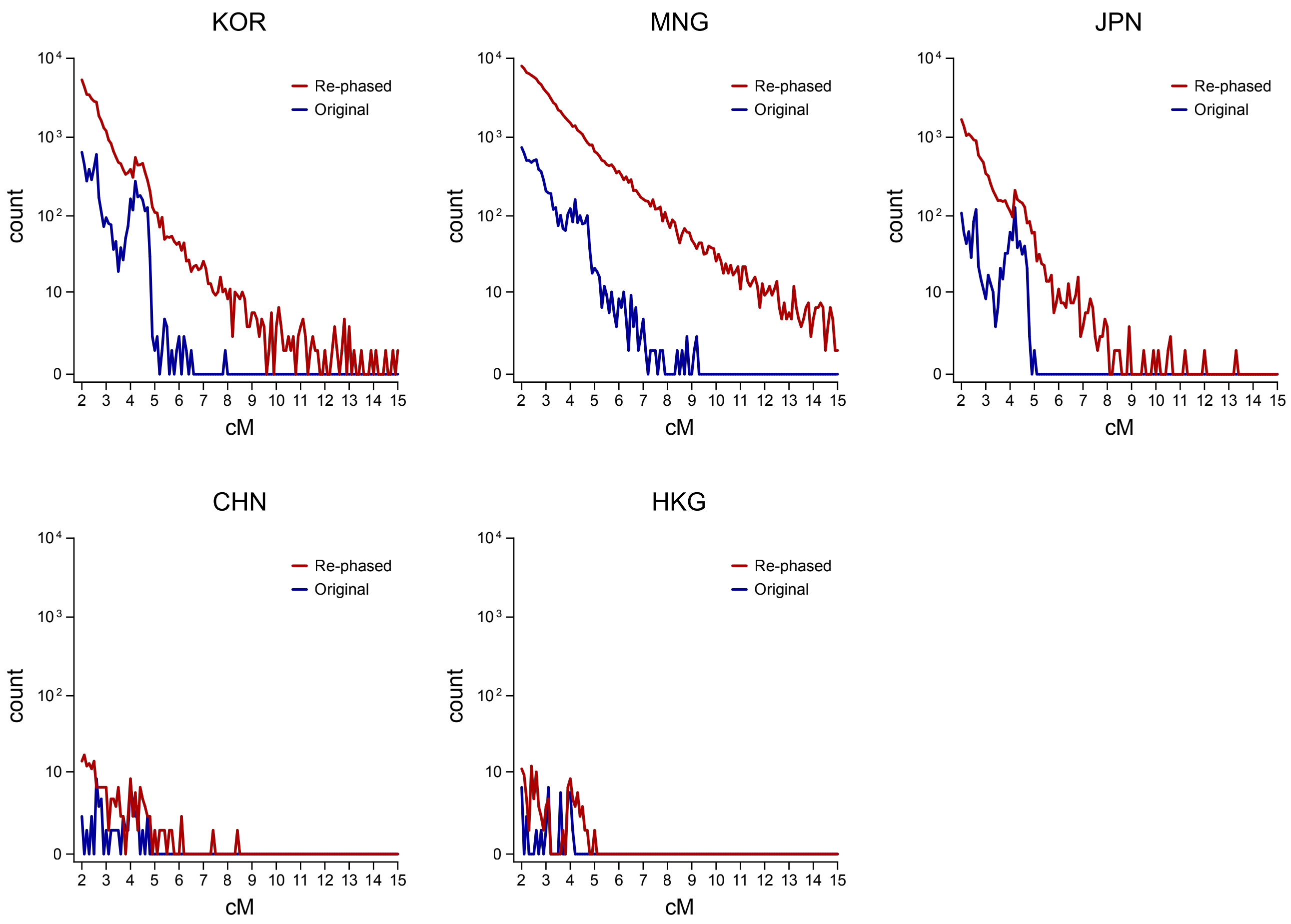

Fig. S11. Length distribution of shared IBD tracts between the two individuals in each population. Distribution of shared IBD tracts which were computed using the original and the re-phased haplotypes were displayed separately. Length of the shared IBDs are different by populations, but consistently increased in the re-phased haplotypes across all.

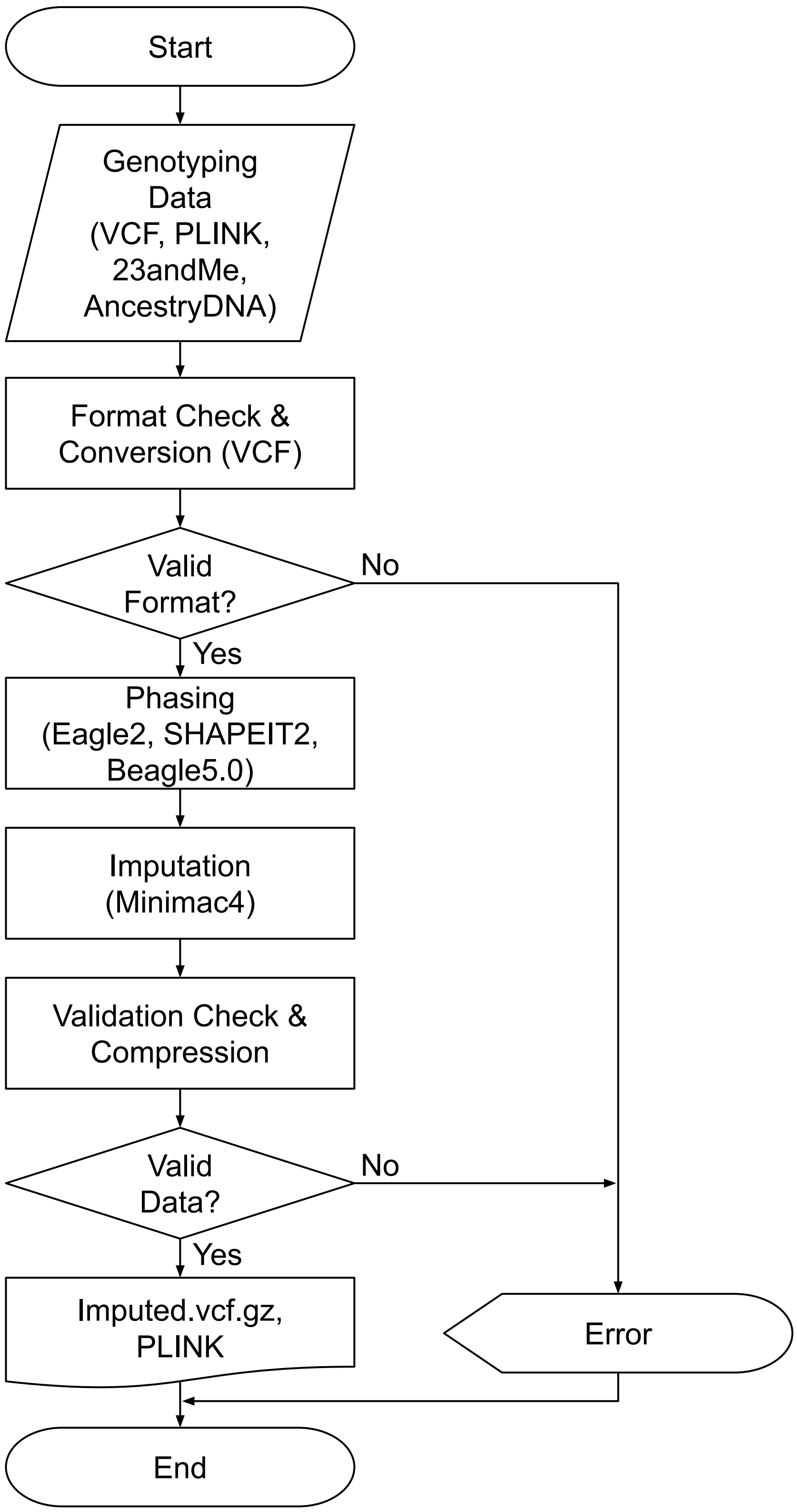

Fig. S12. The flow chart of the pipeline consisting of four major steps for NARD imputation server.

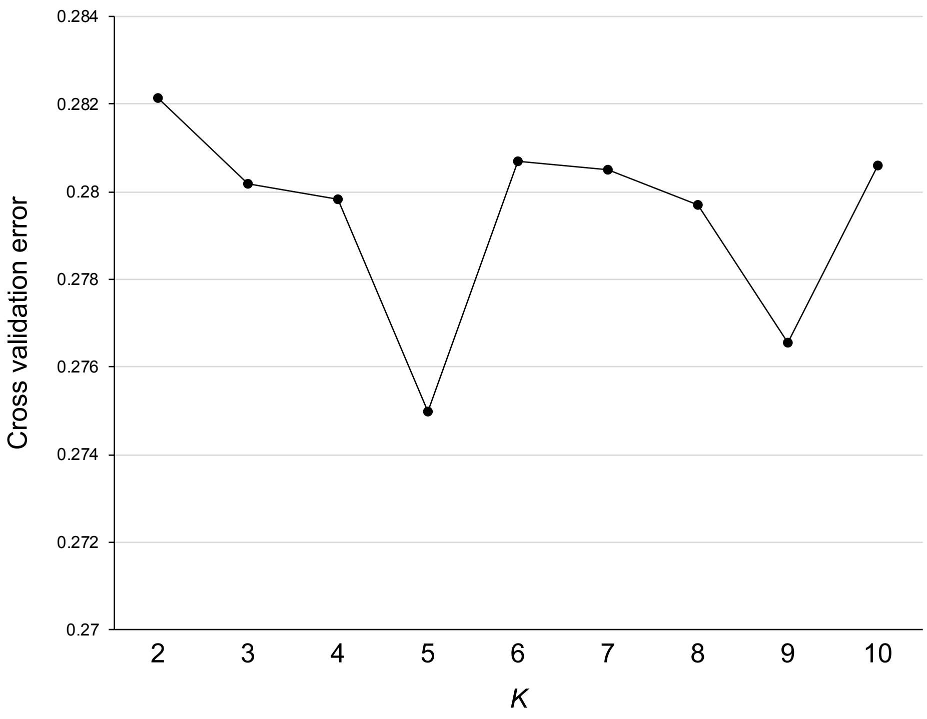

Fig. S13. The cross-validation error inferred by ADMIXTURE algorithm.
